# Editorial: Affective constructs in mathematics education

**DOI:** 10.3389/fpsyg.2024.1373804

**Published:** 2024-02-05

**Authors:** Yusuf F. Zakariya, Adeneye O. A. Awofala, Farzad Radmehr

**Affiliations:** ^1^Department of Science Education, Ahmadu Bello University, Zaria, Nigeria; ^2^Department of Mathematical Sciences, University of Agder, Kristiansand, Norway; ^3^Department of Science Education, University of Lagos, Lagos, Nigeria; ^4^Department of Teacher Education, Norwegian University of Science and Technology, Trondheim, Norway

**Keywords:** emotions, self-efficacy, mathematics anxiety, emotional profiles, motivational profiles

Building on the rejuvenated interest of researchers around the world in affective constructs in mathematics education, we proposed this Research Topic to provide a platform through which mathematics education researchers could express their current thoughts on the topic. Eight manuscripts passed the rigorous review process of Frontiers in Psychology and were published from a pool of manuscripts submitted to the Research Topic. Six of the published manuscripts were original research papers, one was a review paper, and the last one was a brief research report. In an original research paper involving 1,505 elementary school students (ages 6–12), Hanin and Gay characterized the evolution of students' emotional and motivational profiles during mathematical problem-solving tasks. Positive emotions (pride and joy) and negative emotions (anger, boredom, nervousness, and sadness) were prevalent across the 6 years of elementary school with varying intensity depending on grade level and gender. The prevalence of these positive and negative emotions overshadowed that of the anxiety- and self-esteem-related emotional profiles of the students. Each of these emotional profiles was related to students' performance in mathematics, perceived competence, and perceived task value, especially in grades 1, 3, and 6. These findings provide some insight into the types of emotional and motivational profiles and the timing at which targeted interventions will be effective in managing these affective constructs.

In a related study, Prada Núñez et al. characterized the evolution of affective constructs (beliefs, attitudes, and emotions in mathematics) and their relationships with mathematics performance. They found a decline in affective constructs and performance in mathematics across grade levels. This finding is reinforced, albeit in the area of problem-solving understanding, in the brief research report by Sinaga et al. which confirmed that students in the lower grades had higher problem-solving understanding than students in the higher grades. Students in grades 1 up to grade 4 showed positive attitudes toward mathematics, positive beliefs, and positive emotions toward mathematics while demonstrating competence in mathematics. On the other hand, students in grade 11 had low performance, exhibited a loss of interest, had low beliefs, showed and disengaged emotions in mathematics (Prada Núñez et al.). These findings support the hypothesis that “as the student progresses through the various courses of the educational system, he/she loses interest and liking for mathematics, which is reflected in low academic performances and behaviors of apathy and rejection” (SIC) (Prada Núñez et al., p. 8). To provide more explanation for the low academic performance of students, Wu et al. identified test anxiety and parental expectations as exerting negative effects on students' academic performance. These findings are crucial for targeted interventions on anxiety and parent expectations for improved academic performance.

Continuing with the motivational profiles of elementary students, Broda et al. explored this topic and characterized five multidimensional profiles in students' mathematics anxiety, mathematics self-concept, and mathematics interest. These motivational profiles revolved around (a) elevated levels of interest and self-concept coupled with minimal mathematics anxiety; (b) diminished levels of interest and self-concept accompanied by heightened mathematics anxiety; (c) moderate levels of interest, heightened levels of self-concept, and intermediate levels of anxiety. Mathematics interest was found to be a significant predictor of students' engagement in challenging tasks and mathematics self-concept, and it is associated with high prior mathematics knowledge. A similar pattern of association was also reported for mathematics self-concepts with other motivational constructs. These findings provide a complementary perspective to findings reported by Hanin and Gay and Prada Núñez et al. on the emotional and motivational profiles of elementary students. In the context of technology-enhanced learning environments (TELE), students' motivational and emotional profiles played a substantial role. This claim is the hallmark of the original research report by Bednorz and Bruhn. Based on whether students are motivated self-learners, non-motivated self-learners, or averagely motivated non-self-learners, the authors found significant differences in their perceptions of TELE. A key takeaway from the aforementioned studies is that the characterization of students' motivational and emotional profiles is an emerging area of interest in mathematics education.

At the heart of this Research Topic is mathematics self-efficacy, which is defined as “a self-evaluation of students' competence about the presented mathematics tasks that constitutes an internal drive for the successful completion of the task” [SIC] (Zakariya, p. 2). Following a systematic review of previous studies, Zakariya explored interventions that foster mathematics self-efficacy and argued for their implications in mathematics courses. He classified these interventions based on whether sources of self-efficacy were manipulated and whether self-efficacy features were embedded in either instructional methods or learning strategies during the development of these interventions. Further, he provided a step-by-step guide for replicating some of these interventions to improve learning outcomes in mathematics. Focusing on the design of mathematical tasks for improved self-efficacy, Herset et al. found that students reported a high sense of self-efficacy when they worked on tasks marked as easy, medium, and difficult as compared to unmarked mathematics tasks. Their study provides tentative evidence for the effectiveness of using level-marked tasks as a proxy for increasing students' mathematics self-efficacy. In conclusion, the studies in this Research Topic provide state-of-the-art evidence on the relationships within and between affective constructs and other factors that are of great relevance to the teaching and learning of mathematics at all levels of education. We invite you to read each of these studies, replicate their findings, and engage with relevant stakeholders for their implementation.

## Author contributions

YZ: Writing—original draft, Writing—review & editing. AA: Conceptualization, Methodology, Validation, Visualization, Writing—original draft, Writing—review & editing. FR: Writing—original draft, Writing—review & editing.

